# Segmental Normalisation of Lumbar Lordosis Following Transforaminal Lumbar Interbody Fusion for Low-Grade Isthmic Spondylolisthesis

**DOI:** 10.7759/cureus.103827

**Published:** 2026-02-18

**Authors:** Ahmed Elmahdi, Mohamed Youssef, Thomas Robinson, Timothy Boddice, Rajesh Shah

**Affiliations:** 1 Trauma and Orthopaedics, Salford Royal NHS Foundation Trust, Manchester, GBR; 2 Orthopaedics, Hull Royal Infirmary, Hull, GBR; 3 Trauma and Orthopaedics, Hull Royal Infirmary, Hull, GBR

**Keywords:** isthmic spondylolisthesis, lumbar lordosis, sagittal alignment, spinopelvic parameters, transforaminal lumbar interbody fusion (tlif)

## Abstract

Background

Isthmic spondylolisthesis is commonly associated with back pain and neurological symptoms. The primary localised kyphotic deformity at the level of the spondylolisthesis is counterbalanced by increased lordosis across the lumbar spine. Spondylolisthesis reduction and fusion corrects the initial deformity and may also restore lumbar lordosis and sacral slope. In this study, we aimed to investigate how lumbar lordosis normalises following short-segment transforaminal lumbar interbody fusion (TLIF) for isthmic spondylolisthesis.

Methodology

In total, 54 consecutive patients from a single surgeon series of isthmic spondylolisthesis undergoing reduction and TLIF performed between 2013 and 2023 underwent retrospective radiological analysis by two independent observers. Measurements of the lumbar lordosis, sagittal cobb angle across the lumbar spine as a whole, and individual motion segments were taken using pre and postoperative standing radiographs.

Results

A total of 39 fusions were performed at L5/S1, 12 at L4/5, two on both levels L5/S1 and L4/5, and one at L3/4. Normalisation of lordosis was noted at all spinal levels, including those distant from the surgical site. Global lumbar lordosis decreased from a median of 66° to 50° (p < 0.001). All segmental levels showed significant reductions in lordotic angle (p < 0.05), with the greatest proportional change at L1/2 and the largest angular correction at L5/S1.

Conclusions

This study is the first to demonstrate that surgical reduction of isthmic spondylolisthesis can restore global sagittal harmony by correcting the compensatory hyperlordosis across all lumbar segments.

## Introduction

Lumbar back pain is very common in the adult population, with recent estimates of its prevalence of up to 20% [1]. A clear association has been demonstrated between altered sagittal balance and low back pain. In the context of isthmic spondylolisthesis, this often manifests as a compensatory hyperlordosis that develops in response to the local kyphotic deformity at the level of the slip [2].

Isthmic lytic spondylolisthesis affects approximately 5-7% of the population [3] and may be a significant source of back pain in younger patients. The typical deformities are a localised kyphosis at the level of the spondylolisthesis, which is then compensated for by a generalised hyperlordosis to maintain the overall sagittal balance. Such a derangement in sagittal balance increases the rate of fatigue and deformity progression, as well as propagating the progression of arthritic changes in both the hips and facet joints owing to a prolonged abnormal posture [2].

The effect of spinal deformity on the lumbar spine, sacrum, and hips has been well described and categorised by the driving force of the deformity and the means of compensation. ‘Spine-hip syndrome’ is known to create a deleterious compensatory mechanism in the hips driven by the underlying abnormality in the lumbopelvic complex, while ‘hip-spine syndrome’ is described as abnormal hip function driving the deterioration of the lumbar spine [4].

High-grade isthmic lytic spondylolisthesis may be treated with reduction and fusion when conservative measures are unsuccessful; this is considered to be the gold standard in operative management [5]. Correction of the spondylolisthesis deformity is associated with an improvement in symptoms, but there is limited evidence examining the relationship between the reduction of isthmic lytic spondylolisthesis and the pattern of restoration of a normal lumbar lordosis. We aimed to evaluate whether, through the operative reduction of isthmic lytic spondylolisthesis using transforaminal lumbar interbody fusion (TLIF), both the localised kyphosis and the associated generalised hyperlordosis can be corrected, improving the overall sagittal balance.

Hresko et al. described the effect of spondylolisthesis on the sagittal balance throughout the lumbar spine and pelvis [6]. They introduced the concept of the balanced pelvis, in which the compensatory deformity secondary to the spondylolisthesis was borne predominantly by the lumbar spine, leading to an increased lumbar lordosis with a low pelvic tilt (PT) and a high sacral slope (SS). Consequently, the retroverted, unbalanced pelvis was defined as having a higher PT and lower SS, with the compensation for the deformity borne by the pelvis [7].

Shi et al. studied how spondylolisthesis correction using TLIF differed in clinical and radiological outcomes when patients were categorised preoperatively as having either a balanced or an unbalanced pelvis. They demonstrated that operatively reducing the spondylolisthesis through fusion brought about two distinct patterns of change according to the initial group. Corrections in PT were seen when the pelvis was initially unbalanced, while corrections in lordosis alone were seen when the pelvis was initially balanced. Quality of reduction was important and correlated significantly with clinical and radiological outcomes [7].

Despite a thorough review of the literature, we could find no studies that clearly defined or mapped the changes seen when the typical hyperlordotic deformity associated with isthmic lytic spondylolisthesis is corrected through TLIF, motion segment by motion segment across the lumbar spine, and we aimed to evaluate this. Therefore, this study aimed to characterise global and segmental radiographic changes in lumbar lordosis following TLIF for isthmic spondylolisthesis, with particular focus on how correction at the index level influences adjacent lumbar segments. This study was designed as a radiographic analysis and was not intended to correlate alignment changes with validated patient-reported outcome measures.

Normal lumbar lordosis is challenging to define and has been discussed several times in the literature. Vialle et al., in 2005, quoted an average of 43° with a wide variation up to a maximum of 69° [8]. Hay et al., in 2015, investigated the differences between lordosis in males and females and concluded that there was a greater curvature seen in the female spine, associated with greater cranial peak height [9]. Celestre et al., in 2018, suggested that a patient-specific approach should be taken to normal or abnormal lumbar lordosis, but postulated that the population range could be anywhere between 20° and 70° [10].

For the purposes of our study, we defined the normal lumbar lordosis as between the superior endplate of L1 and the superior endplate of S1, with a value of between 30° and 60°. We acknowledge that lumbar lordosis is influenced by pelvic incidence and individual spinopelvic morphology; therefore, this range was used as a pragmatic radiographic reference rather than an absolute alignment target. For this study, ‘normalisation’ was defined as a postoperative lumbar lordosis that fell within the physiologically accepted range of 30°-60°, as described by Roussouly and Nnadi and Vialle [8,11]. Patients whose postoperative lumbar lordosis values remained outside this range were considered not to have achieved full radiographic normalisation.

## Materials and methods

Study design and patient selection

A retrospective cohort study was conducted among 54 patients who underwent TLIF for isthmic lytic spondylolisthesis between 2013 and 2023. Although the study primarily focused on low-grade (Grade I-II) isthmic spondylolisthesis, a small proportion of Grade III cases were included as part of a consecutive single-surgeon series following ethical approval from our local audit and governance department. Patients with prior lumbar surgery, multi-level fusion extending beyond the index isthmic level, or significant coronal deformity were excluded. Preoperative and postoperative radiographic parameters were analysed to assess changes in lumbar lordosis, SS, and residual spondylolisthesis. Postoperative measurements were obtained from the first standing lateral radiograph performed approximately six weeks after surgery. This time point was chosen to represent early functional sagittal alignment rather than intraoperative positioning. Patients were reviewed clinically for a mean follow-up of 12 months following surgery. In addition, patient demographics and clinical outcomes, including intra/postoperative complications and persistent symptoms, were recorded. Clinical status was determined through routine outpatient follow-up documentation rather than validated patient-reported outcome measures (PROMs). The indications for surgical intervention were failure of non-operative treatment or evidence of worsening neurological compromise. No patients in this series underwent surgery based solely on radiological criteria.

Surgical technique

All patients underwent TLIF in a prone position through a bilateral Wiltse paraspinal approach. All cases were performed open using a posterior pedicle screw instrumentation system. No cases were performed minimally invasively. Image intensifiers were used to site pedicle screws bilaterally before distraction, decompression, reduction, and interbody TLIF cage filled with morsellised autograft bone harvested from the posterior elements being sited. Final compression was performed to aid arthrodesis and restore alignment. Static lordotic TLIF cages were used in all cases, with cage lordotic angles ranging from 10° to 13° depending on intraoperative alignment following instrumented correction. Segmental lordosis was primarily achieved through pedicle screw instrumentation and reduction manoeuvres rather than cage geometry. The cages were inserted to span the full anteroposterior length of the disc space, extending from the anterior longitudinal ligament to the posterior longitudinal ligament to provide anterior column support. There was no variation in cage positioning between L5/S1 and L4/5 levels.

Radiographic Measurements

**Figure 1:**
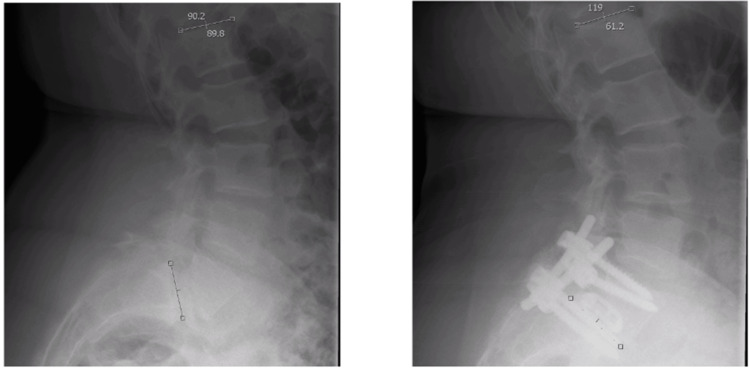
Change in lumbar lordosis.

**Figure 2:**
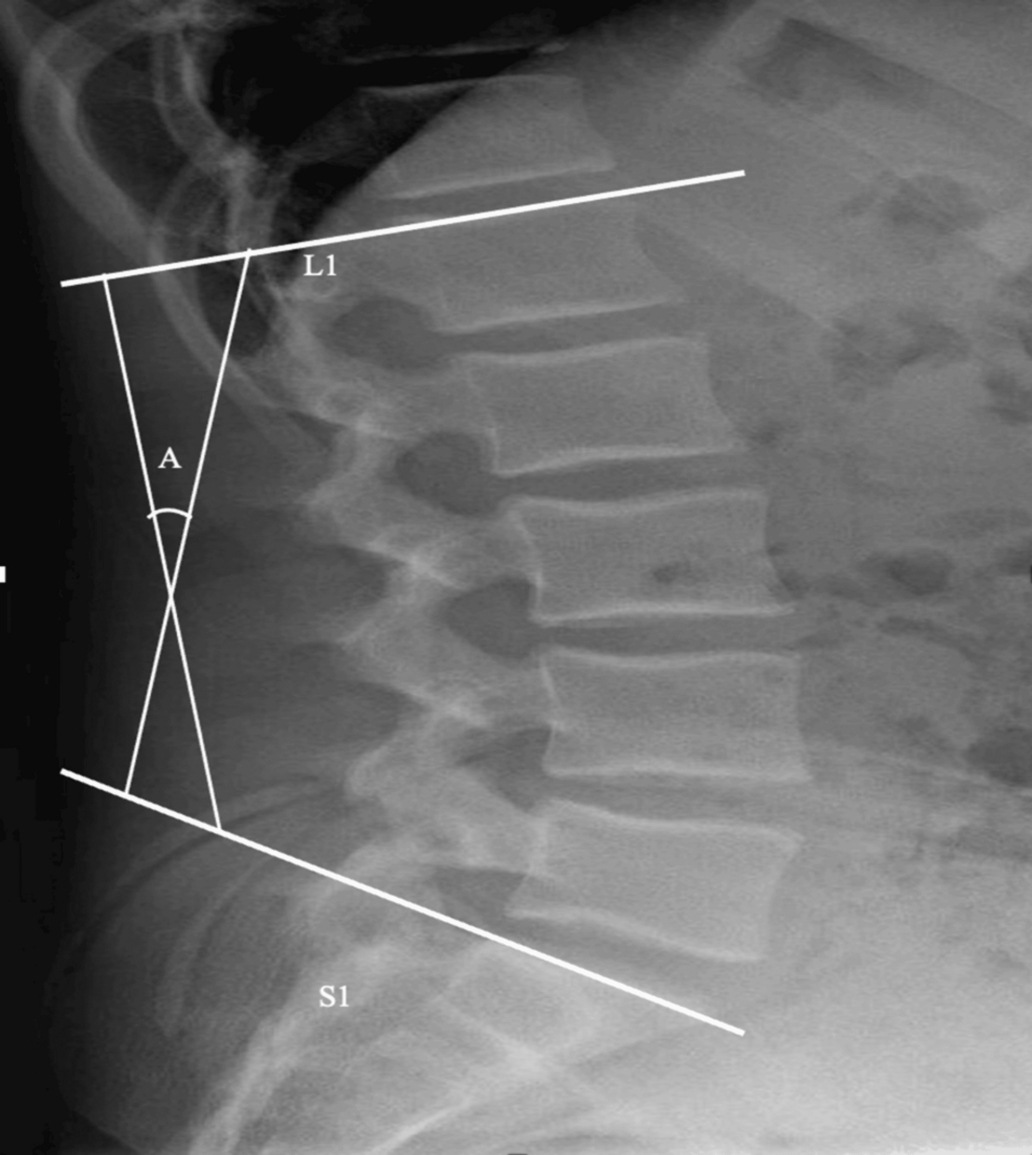
Measuring lumbar lordosis.

**Figure 3:**
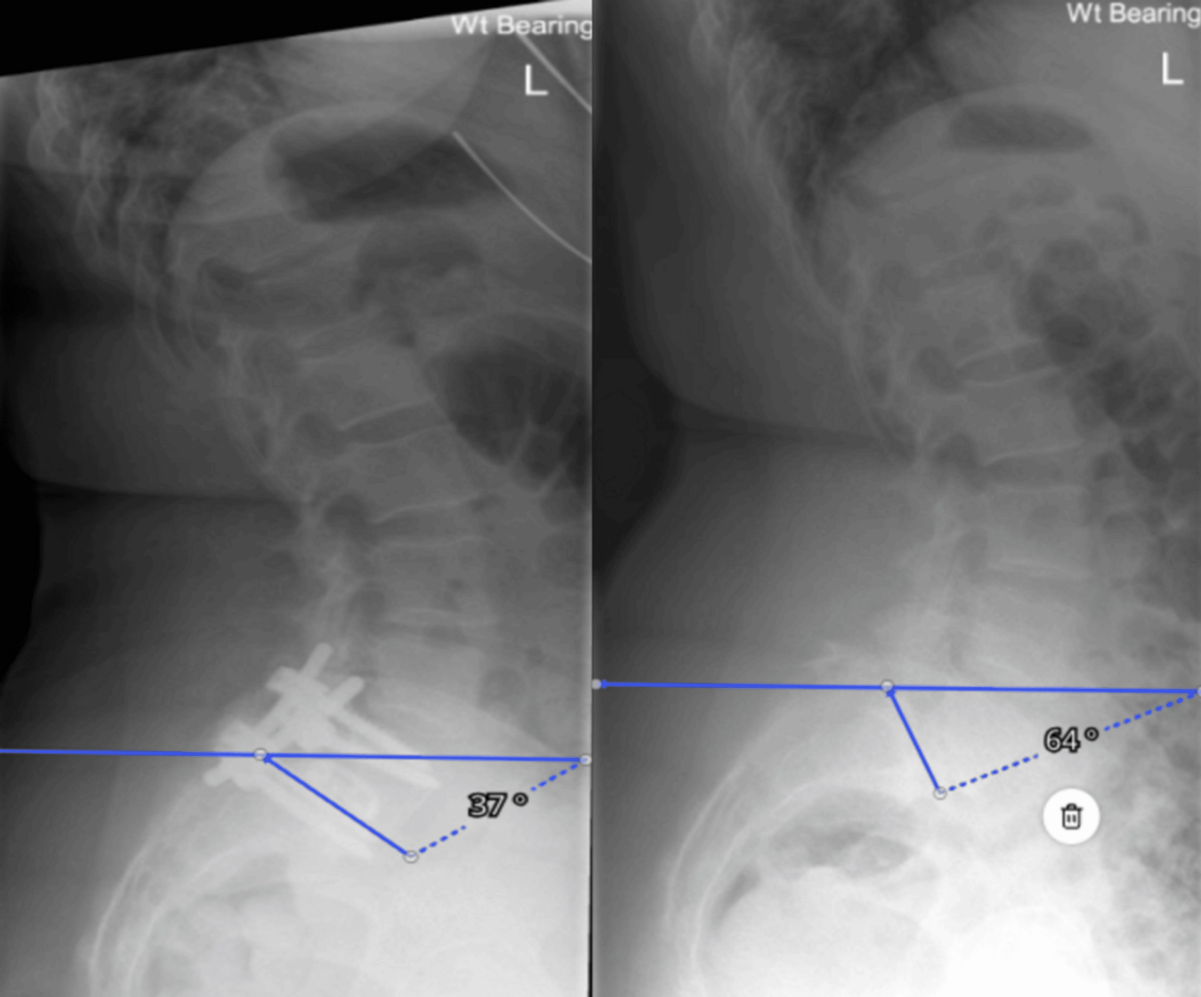
Measuring the sacral slope.

Pre and postoperative standing lateral radiographs were analysed by two independent assessors, and angles were measured and recorded (Figure 1). Lumbar lordosis was measured between the superior endplate of L1 and the superior endplate of S1 (Figure 2). SS was measured between the tangent line to the superior endplate of S1 and the horizontal plane (Figure 3). Radiographs were viewed using PACS software, and angles were measured using the PACS angle measurement tool. To evaluate measurement reliability, 35% of radiographs were independently re-measured by both observers for lumbar lordosis, SS, and L5/S1 segmental angles. Inter-observer agreement was assessed using a two-way random-effects, absolute-agreement intraclass correlation coefficient (ICC(2,1)), and intra-observer repeatability (same observer, ≥2 weeks apart) using a two-way mixed-effects ICC (ICC(3,1)). Both analyses demonstrated excellent reliability (ICC > 0.85 for all parameters), confirming the consistency and reproducibility of the radiographic measurements.

Statistical analysis

Normality of pre and postoperative radiographic measurements was assessed using the Shapiro-Wilk test. Although most levels were normally distributed, the L1/2 segment violated normality assumptions. All radiographic comparisons were analysed using the non-parametric Wilcoxon signed-rank test. Radiographic values are reported as medians with first (Q1) and third (Q3) quartiles. Effect sizes were calculated as r (z ÷ √N). A p-value <0.05 was considered statistically significant.

## Results

A total of 54 patients who underwent TLIF for isthmic lytic spondylolisthesis were included in this study. The mean age was 48 ± 12 years (range = 24-72 years). There were 28 (51%) males and 26 (48%) females. The majority had an American Society of Anesthesiologists (ASA) score of 2 (31 patients, 57%), followed by ASA 3 (13 patients, 24%) and ASA 1 (10 patients, 19%). Most patients had Grade II spondylolisthesis (41 patients, 76%), while Grade III and Grade I were observed in seven (13%) and six (11%) patients, respectively. The most commonly affected level was L5/S1 (39 patients, 72%), followed by L4/5 (12 patients, 22%), with two (4%) patients having spondylolisthesis at both L4/5 and L5/S1, and one (2%) patient at L3/4. The majority of cases underwent one-level fusion (49 patients, 91%), while five (9%) patients had two-level fusion. The mean Charlson Comorbidity Index (CCI) was 2 ± 2 (range = 0-6) (Table 1).

**Table 1:** Baseline demographic and clinical characteristics of the study population (n = 54). ASA = American Society of Anesthesiologists

Characteristic	Value
Total patients	54
Age, mean ± SD (range)	48 ± 12 (24–72)
Male sex, n (%)	28 (51%)
Female sex, n (%)	26 (48%)
ASA I, n (%)	10 (19%)
ASA II, n (%)	31 (57%)
ASA III, n (%)	13 (24%)
Charlson Comorbidity Index, mean ± SD (range)	2 ± 2 (0–6)
Spondylolisthesis Grade I, n (%)	6 (11%)
Spondylolisthesis Grade II, n (%)	41 (76%)
Spondylolisthesis Grade III, n (%)	7 (13%)
Affected level L3/4, n (%)	1 (2%)
Affected level L4/5, n (%)	12 (22%)
Affected level L5/S1, n (%)	39 (72%)
Affected levels L4/5 and L5/S1, n (%)	2 (4%)
One-level fusion, n (%)	49 (91%)
Two-level fusion, n (%)	5 (9%)

Radiographic outcomes

**Figure 4:**
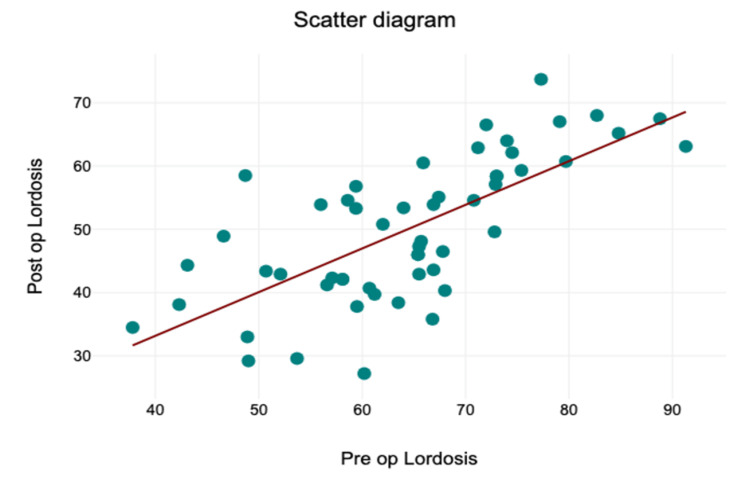
Scatter diagram showing change in lordosis.

Preoperative global lumbar lordosis (L1-S1) had a median of 66° (IQR = 58-73°), which significantly decreased to 50° (IQR = 42-59°) postoperatively (W = 1466, z = 6.23, p < 0.001, r = 0.85) (Figure 4).

A significant reduction was also demonstrated at each individual lumbar level (Table 2). The greatest angular correction occurred at L5/S1, while the largest proportional change occurred at L1/2, reflecting global relaxation of compensatory hyperlordosis. Sacral slope decreased in line with these changes, consistent with restoration of lumbosacral alignment.

**Table 2:** Segmental and global lumbar lordosis before and after TLIF for isthmic spondylolisthesis. TLIF = transforaminal lumbar interbody fusion

Segment	Preoperative lordosis, median (°)	Q1–Q3	Postoperative lordosis, median (°)	Q1–Q3	W	Z	P-value	Effect size (R)
L1-S1 (global)	66°	58°–73°	50°	42°–59°	1,466	6.23	<0.001	0.85
L5/S1	22°	16°–28°	18°	13°–22°	1,242.5	4.31	<0.001	0.59
L4/5	16°	12°–20°	13°	11°–16°	1,116	3.22	0.001	0.44
L3/4	12°	9°–14°	10°	7°–13°	1,178.5	3.75	<0.001	0.51
L2/3	8°	6°–11°	7°	3°–10°	938	1.97	0.049	0.27
L1/2	4°	2°–6°	2°	1°–4°	1,058	3.03	0.002	0.41

Complications and clinical outcomes

A total of nine (17%) patients experienced postoperative complications. The most common complications included spontaneous pneumothorax (two patients), altered sensation (two patients), and perineural fibrosis (two patients), while infection (one patient), screw breach (one patient), and foraminal stenosis (one patient) were also observed. Persistent symptoms were reported in nine (17%) patients, while 45 (83%) patients were symptom-free postoperatively.

There was no significant difference in lordosis correction between one-level and two-level fusion (Wilcoxon signed-rank test, p = 0.76). Similarly, L5/S1 versus L4/5 surgeries did not significantly differ in lordosis correction (p = 0.72). Residual spondylolisthesis had a median of 2.6 mm (IQR = 0.9-4.8 mm) and was not significantly associated with persistent symptoms (p = 0.85) or complications (p = 0.85) (Table 3).

**Table 3:** Residual spondylolisthesis following surgical reduction. IQR = interquartile range

Residual category	n	%
<2 mm	25	46%
2–4 mm	12	22%
>4 mm	17	31%
Total	54	100%
Residual listhesis (mm), median (IQR)	2.6 (0.9–4.8)	

Logistic regression models showed no significant predictors of complications (χ² = 4.52, p = 0.34) with p-values >0.05 for all variables, although age (p = 0.083) and preoperative lordosis (p = 0.518) showed weak trends (Table 4).

**Table 4:** Logistic regression analysis of predictors of postoperative complications.

	Odds ratio	95% confidence interval	P-value
Constant	0.81	0–137.04	0.937
Age	1.07	0.99–1.16	0.083
Sex, female	1.67	0.34–8.15	0.526
Charlson Comorbidity Index	1.03	0.63–1.67	0.905
Preop lordosis	0.98	0.91–1.05	0.518
Residual listhesis (mm)	1.02	0.86–1.20	0.85
Model fit (likelihood ratio χ², df = 4)	-	-	χ² = 4.52, p = 0.34

Similarly, predictors of persistent symptoms were not statistically significant (χ² = 5.19, p = 0.268), with age (p = 0.885), sex (p = 0.311), CCI (p = 0.133), and preoperative lordosis (p = 0.581) all showing no significant associations (Table 5).

**Table 5:** Logistic regression analysis of predictors of persistent symptoms.

	Odds ratio	95% confidence interval	P-value
Constant	0.05	0–6.16	0.222
Age	1.01	0.93–1.08	0.885
Sex, female	2.29	0.46–11.42	0.311
Charlson Comorbidity Index	0.6	0.31–1.17	0.133
Preoperative lordosis	1.02	0.95–1.09	0.581
Residual listhesis (mm)	1.04	0.86–1.26	0.703
Model fit (likelihood ratio χ², df = 4)	-	-	χ² = 5.19, p = 0.268

Residual spondylolisthesis did not significantly predict persistent symptoms (χ² = 0.04, p = 0.851). The odds ratio (OR) for residual spondylolisthesis predicting persistent symptoms was 1.02 (95% confidence interval (CI) = 0.86-1.2, p = 0.85), indicating no meaningful association. Even after adjusting for age, sex, and CCI, residual spondylolisthesis remained a non-significant predictor of persistent symptoms (χ² = 5.03, p = 0.285), with an adjusted OR of 1.04 (95% CI = 0.86-1.26, p = 0.703).

## Discussion

The most prominent finding from our analysis comparing preoperative to postoperative measurements of lumbar lordosis following single- or double-level TLIF for isthmic lytic spondylolisthesis was a normalisation of the patients’ lordosis across all levels in the lumbar spine, not simply at the operative site. Our expectation that the greatest angular change contributing to the normalisation of hyperlordosis would be seen at the operative level was borne out through our data; yet, the greatest proportional change was shown to be at L1/2 in all patients in whom the lordosis normalised. At L1/2, median lordosis decreased from 4° preoperatively to 2° postoperatively, representing a 50% relative reduction, which illustrates the distal-to-proximal relaxation effect observed following reduction at the lumbosacral junction. This supports the hypothesis that through the correction of the primary deformity, the patients’ overall sagittal balance globally improves throughout the entire lumbar spine, even in motion segments distant from the surgical site. At first glance, the reduction of a kyphotic spondylolisthesis might be expected to increase rather than decrease lumbar lordosis. However, the opposite was observed in our series, which reflects the correction of compensatory, not physiological, lordosis. Preoperatively, the anterior translation and kyphotic angulation at the level of the slip shift the trunk’s centre of gravity anteriorly. To maintain sagittal balance and an upright posture, the lumbar segments above the spondylolisthesis hyperextend, producing a global hyperlordosis. Once the local kyphotic deformity is reduced and the lumbosacral alignment restored, this compensatory hyperextension is no longer required. Consequently, the total lumbar lordosis decreases towards a normal value, a finding that represents radiological normalisation rather than loss of curvature.

To our knowledge, previous studies have focused primarily on local slip reduction and fusion alignment, without mapping the resulting changes across the entire lumbar spine. Our analysis demonstrates, for the first time, that reduction of isthmic spondylolisthesis leads to a proportional decrease in compensatory hyperlordosis at levels distant from the operative site. This suggests that restoring segmental alignment at the lumbosacral junction can rebalance the lumbar curvature as a whole, achieving a more harmonious physiological profile. This proximal reduction in compensatory hyperlordosis across non-operated segments represents the radiographic basis of the ‘segmental normalisation’ described in our title.

The largest angular correction at the L5/S1 level similarly reflects restoration of physiological alignment rather than a measurement artefact. Reduction of the slip realigns the L5 vertebral body over the sacrum and decreases the sacral slope, effectively flattening the previously exaggerated local curvature. This reduction in the L5/S1 segmental angle, therefore, denotes correction of a pathological compensatory lordosis rather than a loss of normal segmental lordosis.

All except two patients in our single-surgeon series had a decrease in their overall radiologically measured lumbar lordosis postoperatively. Of these two patients who did not follow the overall trend, one patient’s lumbar lordosis measured 47° preoperatively and postoperatively measured 49°, starting and finishing within our accepted normal limits. They had an uneventful recovery and were discharged at one year. The second patient preoperatively measured 49° and postoperatively measured 64° on standard weight-bearing radiographs, taking them from within our accepted normal range to 4° above our defined upper limit, a change of +15°. This was reviewed by the senior author, and no obvious cause could be identified. The slip was reduced to within 3.5 mm, and the fixation was appropriately placed. The patients’ overall standing posture on the weight-bearing radiographs appears to be somewhat abnormally extended, nearly mimicking a formal lumbar extension radiograph. They clinically made a full symptomatic recovery, being discharged with no back or leg pain at one year.

Lenke et al.’s work in the 1990s showed that despite non-anatomical reduction of their isthmic spondylolisthesis, around 80% of their patients’ preoperative symptoms improved with in situ fusion, even if the grade of the fusion was radiographically uncertain [12] and the sagittal balance left unresolved, but the slip was stabilised. Yet, Harroud et al. found an association between deviation of sagittal alignment and poorer pain scores in a young population presenting with isthmic spondylolisthesis [13], drawing attention to the importance of sagittal malalignment, especially in higher-grade slips in a younger population. This is further supported by Boachie-Adjei et al., who proposed that postoperative improvements are linked to reduction of the localised kyphotic deformity commonly associated with spondylolisthesis, which restores the sagittal axis, as opposed to reduction of the percentage of slip [14]. It is the mechanism by which this correction of the localised kyphotic deformity is able to normalise lumbar lordosis throughout the lumbar spine that we have sought to map out and develop an understanding of in our study.

Our study describes the pattern of normalisation of lumbar lordosis seen postoperatively in patients undergoing TLIF for isthmic lytic spondylolisthesis. Lumbar lordosis has been shown to be a radiographic parameter that, when normalised, is significantly correlated with improved recovery rates and reduction in the incidence of severe back pain in the existing literature [5,7,13,14]. Understanding the anatomical impact of single- or double-level fusion on the lumbar spine as a whole, even in motion segments distant from the surgical site, allows us to better appreciate how correction of the typically encountered patho-anatomy works level by level to restore a ‘normal’ sagittal balance, as well as helping us understand how this may affect clinical outcomes.

Our findings also align with emerging comparative evidence regarding the surgical approach in TLIF. Recent studies have shown that the essential determinant of sagittal realignment is not whether the procedure is performed through an open or minimally invasive corridor, but rather the achievement of adequate slip reduction and restoration of disc height. Zhang et al. [15] demonstrated that both minimally invasive surgery (MIS) and open TLIF produce comparable improvements in segmental and regional lordosis, despite MIS offering advantages in blood loss and soft-tissue disruption. Similarly, Li et al. [16] reported no significant differences in the magnitude of radiological correction between MIS and mini-open TLIF for low-grade spondylolisthesis. These data support our interpretation that the global normalisation of lumbar lordosis observed in our cohort is primarily driven by successful reduction of the deformity rather than the surgical approach itself. While our series reflects an open TLIF cohort, and we acknowledge the distinct perioperative profile of this technique, our radiological outcomes confirm that the core biomechanical goals of deformity correction are reliably achieved.

Our study has also illustrated how the SS is reduced following the reduction of the spondylolisthesis and normalisation of lumbar lordosis. Intraoperatively, this was not a primary objective, but we feel this is a result of the reduction of the spondylolisthesis itself. It is our opinion, therefore, that the sacrum changes its orientation in relation to the L5 vertebral body. This change in sacral slope accounts for the slight reduction in the L5/S1 lordosis seen following reduction of the slip. We did not measure pelvic incidence (PI) or PT. As PI is a fixed anatomical parameter, changes in SS would theoretically influence PT; however, this relationship was not directly assessed in our study, and therefore any inferred effect on spinopelvic alignment should be interpreted with caution.

Our observations are consistent with those of Vialle et al., who reported that patients with isthmic spondylolisthesis commonly exhibit a compensatory pattern characterised by increased lumbar lordosis and elevated sacral slope. Their 2022 study demonstrated that correction of the primary translational deformity leads to progressive normalisation of these parameters, reflecting a return towards physiological sagittal balance [17]. This aligns directly with our finding that hyperlordosis across multiple lumbar segments decreases following reduction of the slip, supporting the view that these changes represent relaxation of compensatory mechanisms rather than loss of normal curvature.

Similarly, Wang et al. analysed sagittal parameters in isthmic spondylolisthesis and highlighted the close association between slip severity, disc height collapse, and compensatory hyperlordosis [18]. Their work showed that restoration of disc height and reduction of vertebral translation produce predictable improvements in segmental and global lordosis. These findings reinforce our interpretation that the postoperative harmonisation of lumbar lordosis observed in our cohort is primarily driven by mechanical correction of the underlying deformity rather than changes induced by surgical exposure or fixation technique.

Awareness and understanding of spinopelvic alignment is not only of interest to the spinal surgery community, as arthroplasty surgeons are often confronted with patients with pre-existing sagittal malalignment due to spinal pathology or patients who have undergone prior lumbosacral fixation [19,20]. Developments in the understanding of how sagittal balance influences appropriate acetabular cup orientation, in a bid to prevent dislocation, are ongoing. The work of Sultan et al. showed that the pelvis can no longer be considered an anatomical unit in isolation; rather, spinopelvic anatomy should be considered in concert to enable patient-specific prosthetic orientation to minimise dislocation risk, which has been shown to be higher in patients with adult spinal deformity and abnormal spinopelvic alignment [21].

This study confirms that reduction of isthmic lytic spondylolisthesis produces a global harmonisation of lumbar lordosis, including levels distant from the fusion, reflecting true biomechanical rebalancing rather than isolated segmental change.

Limitations

This study is retrospective in design and based on existing radiographs acquired as part of standard clinical practice. The radiographic field of view did not consistently include the femoral heads, which prevented accurate measurement of certain spinopelvic parameters such as PI and PT. Consequently, the proposed relationship between the observed changes in SS and overall sagittal balance remains inferential rather than directly quantified. A further limitation is the absence of validated PROMs such as the Oswestry Disability Index (ODI) or Visual Analogue Scale (VAS). Because this was a retrospective radiographic analysis, only binary symptomatic data were available, which limited correlation between radiographic normalisation and functional outcomes. Although the cohort was predominantly low-grade, a small proportion of Grade III slips were included, which may introduce heterogeneity in correction patterns. Finally, the relatively small sample size reduces statistical power and limits the generalisability of our findings. Clinical success was determined from documented follow-up assessments rather than standardised PROMs such as ODI or VAS, which limits objective evaluation of functional improvement. Future prospective studies incorporating larger patient cohorts, comprehensive spinopelvic measurements, and standardised PROMs will be essential to fully elucidate the relationship between segmental correction, global alignment, and clinical recovery.

## Conclusions

Reduction of isthmic lytic spondylolisthesis using TLIF results in global harmonisation of lumbar lordosis, including motion segments distant from the operative level. The observed decrease in compensatory hyperlordosis suggests restoration of physiological lumbar sagittal alignment rather than loss of curvature. These findings highlight the biomechanical impact of segmental correction on overall lumbar balance and may have implications for surgical planning in patients with sagittal malalignment.
